# In vitro efficacy of daptomycin and teicoplanin combined with ethanol, clarithromycin or gentamicin as catheter lock solutions

**DOI:** 10.1186/s12866-015-0585-3

**Published:** 2015-10-30

**Authors:** Diego Parra, Alejandro Peña-Monje, Nieves María Coronado-Álvarez, José Hernández-Quero, Jorge Parra-Ruiz

**Affiliations:** Laboratorio de Investigación Antimicrobiana, Hospital Universitario San Cecilio. Instituto de Investigación Biosanitaria de Granada, 18012 Granada, Spain; Servicio de Microbiología, Hospital Universitario San Cecilio, 18012 Granada, Spain; Servicio de Enfermedades Infecciosas, Hospital Universitario San Cecilio. Instituto de Investigación Biosanitaria de Granada, Avda Dr. Olóriz 16, 18012 Granada, Spain

**Keywords:** Biofilm, Central line associated bloodstream infections, Antibiotic lock therapy

## Abstract

**Background:**

Despite widespread use, optimum choice of antimicrobial agents, concentrations, combinations and exposure times have not been determined for antibiotic lock technique (ALT). Our objective was to evaluate the efficacy of different antibiotic combinations using an in vitro model of catheter-related infection. Daptomycin (DAP) 5 mg/mL, teicoplanin (TEC) 5 mg/mL, both alone and combined with gentamicin (GM) 2.5 mg/mL, clarythromycin (CLA) 5 mg/mL or ethanol 35 % were evaluated against four clinical strains of methicillin resistant coagulase negative staphylococci. Lock solutions were renewed every 24 h.

**Results:**

After 72 h catheters were reincubated with culture media to investigate bacterial regrowth. All antibiotic combinations resulted in significant reductions (*p* < 0.05) of Log(10) cfu/mL at 72 h for both organisms compared with controls. DAP resulted in significant reductions of Log(10) for all organism versus TEC (*p* = 0.001). Only DAP reached the limit of detection at 72 h, however did not prevent regrowth after 24 h of ALT removal. DAP + Ethanol and TEC + ethanol eradicated biofilm at 72 h, but only DAP + ethanol (against all strains) and DAP + CLA (against two strains) prevented regrowth at 24 h after ALT removal.

**Conclusions:**

Based on these data, ALT with DAP + ethanol and DAP + CLA should be explored in clinical trials.

## Background

Recent reports indicate that central line associated bloodstream infections (CLA-BSIs) are responsible of considerable burden of illness in many countries, with a pooled prevalence of 4,9 per 1000 central line days in ICUs from Latin America, Asia, Africa and Europe [1]. Many microorganisms can develop biofilms on central venous catheters And therefore be responsible of central line associated bloodstream infections (CLA-BSIs), although the most commonly isolated microorganism from hospital-acquired central line associated bloodstream infections (CLA-BSIs), accounting for almost 35 % of cases, are coagulase-negative staphylococci (CoNS) [2]. Among CoNS, *Staphylococcus epidermidis* (SE) is responsible of a great number of those infections [3]. Pathogenesis of CLA-BSIs has been extensively reviewed; intraluminal or extraluminal surface colonization of catheters, subsequent biofilm formation, dispersal and entry into the bloodstream are the classical steps described in CLA-BSIs development [4–6].

Current guidelines recommend removal of catheters in CLA-BSIs due to *S. aureus* however, antibiotic lock therapy (ALT) could be used in other staphylococcal CLA-BSIs provided sepsis, endocarditis, suppurative thrombophlebitis or other septic complications have been excluded [7, 8]. During ALT the lumen is filled with a highly concentrated antibiotic solution and allowed to dwell (lock) for a period of time to eradicate bacteria embedded in the intraluminal biofilm of the catheter [4]. Since 1980s several authors have reported case series regarding efficacy of ALT against different microorganisms, and although there are some comprehensive reviews on this topic [9, 10], its utility is still a matter of debate. Recent efforts have been made to overcome this paucity of data, but apart from the need of randomized controlled trials no other conclusions have been obtained [11].

Similarly, experimental evidence for the selection of antimicrobials, alone or in combination is scarce and limited to a 24–48 h period without further evaluation of the persistence of biofilm, limiting the interpretation of results [12–17].

To overcome some of these limitations we compared daptomycin and teicoplanin alone, and in combination with clarithromycin, gentamicin and ethanol, using an in vitro catheter model that evaluates their activity in a catheter lock solution by quantifying antimicrobial kill after 72 h of catheter-lock therapy. Both drugs are antibacterial agents with documented efficacy as catheter lock solutions [12, 18–20]. In addition, to evaluate relapse of infection we allowed catheters to dwell without antibiotic for another 24 h, simulating clinical practice of reutilization of central line after ALT.

## Results and discussion

Isolates were susceptible to daptomycin (MIC = 0.25 mg/L for all isolates) and to teicoplanin (MIC = 1 mg/L for 1022 and MIC = 2 mg/L for 1018, 1076, and 1098) and resistant to oxacillin (MIC > 1024 mg/L for all isolates), gentamicin (MIC > 32 mg/L for all isolates) and to clarithromycin (MIC > 128 mg/L for all isolates).

All antibiotic combinations resulted in significant reductions (*p* < 0.05) of Log_10_CFU/mL at 72 h compared with control. Among single-agent regimens, daptomycin resulted in significant reductions of Log_10_CFU/mL versus teicoplanin (*p* = 0.001) for all strains tested, however, neither daptomycin nor teicoplanin were able to reduce Log_10_CFU/mL below the limit of detection at 72 h, and full regrowth was observed after 24 h of ALT removal.

**Fig. 1:**
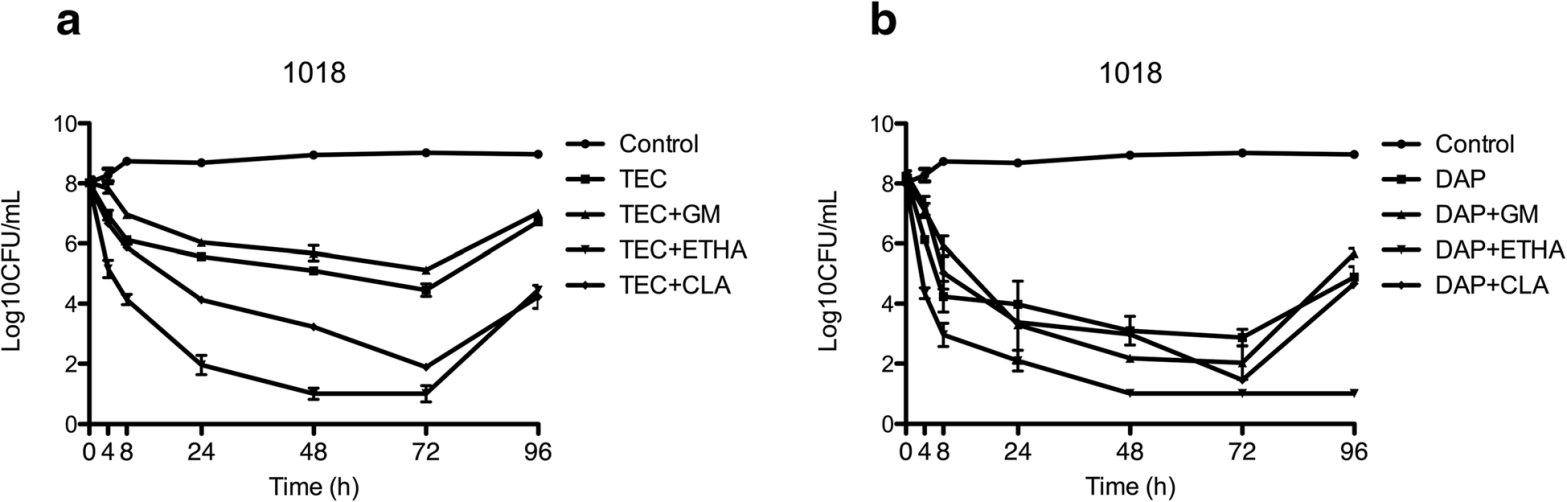
Activity of Teicoplanin (**a**) and daptomycin (**b**) alone and in combination against strain 1018

**Fig. 2:**
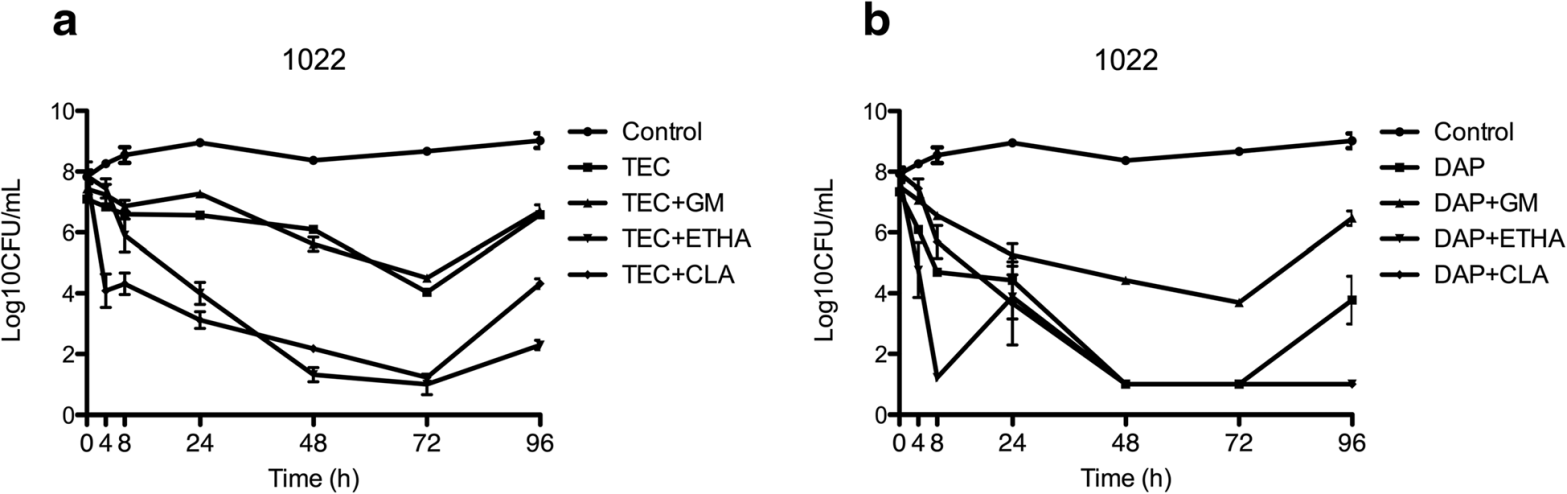
Activity of Teicoplanin (**a**) and daptomycin (**b**) alone and in combination against strain 1022

**Fig. 3:**
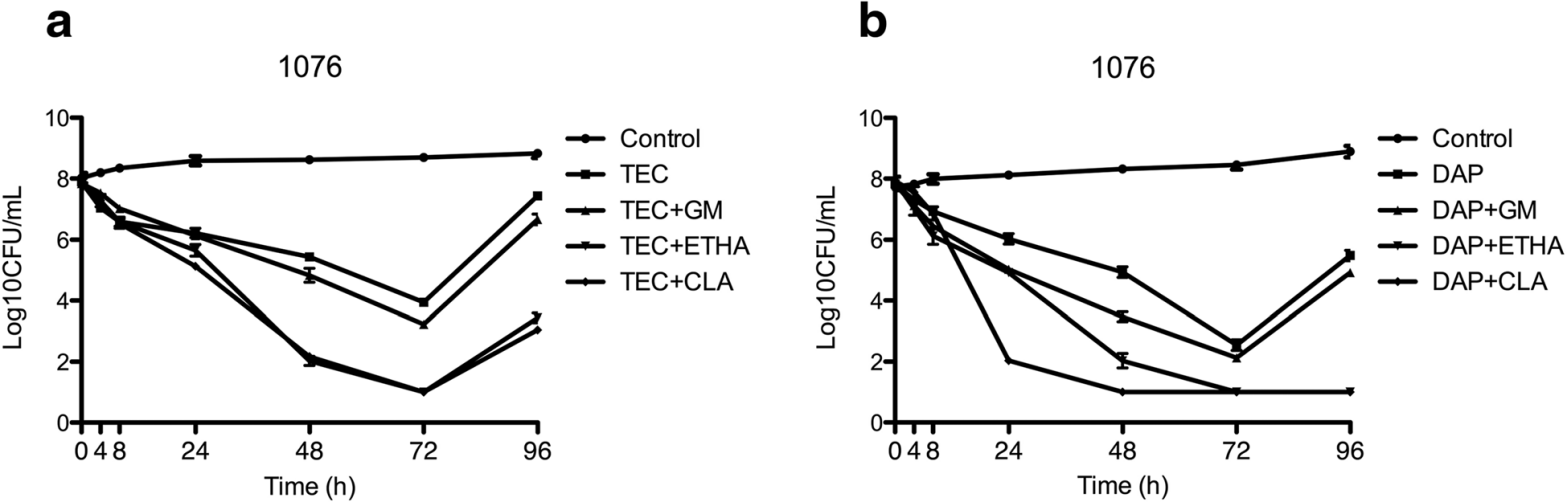
Activity of Teicoplanin (**a**) and daptomycin (**b**) alone and in combination against strain 1076

**Fig. 4:**
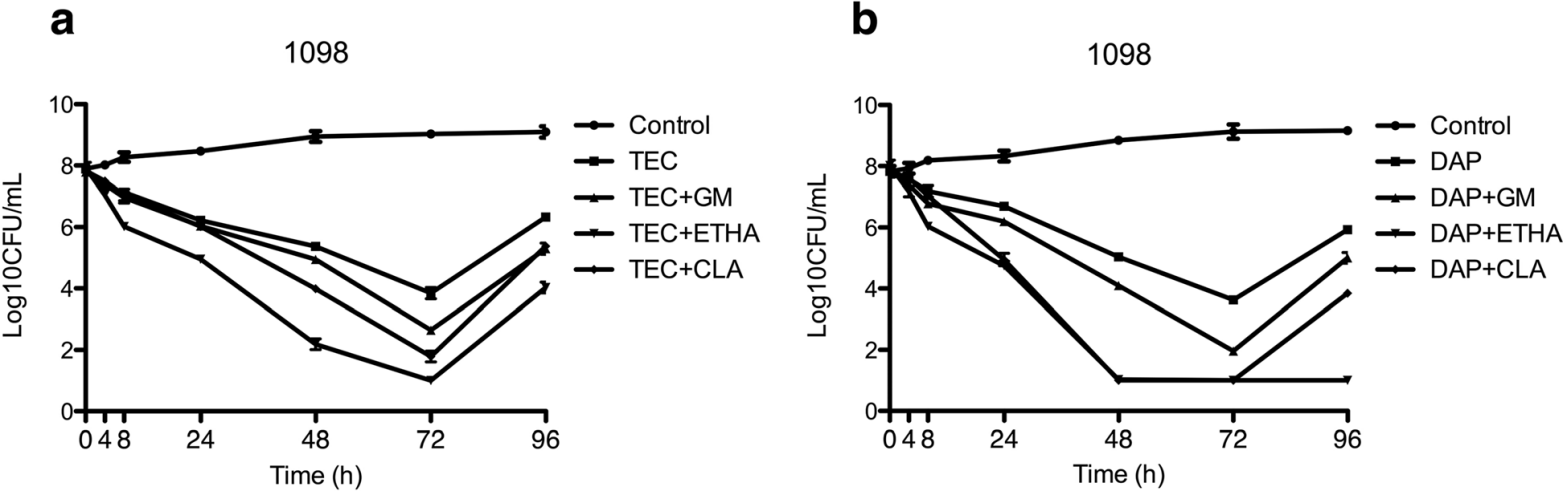
Activity of Teicoplanin (**a**) and daptomycin (**b**) alone and in combination against strain 1098

Regarding combinations, daptomycin plus ethanol or clarithromycin and teicoplanin plus ethanol or clarithromycin were able to eradicate biofilm at 72 h but only daptomycin plus ethanol against all strains and daptomycin plus clarithromycin against two strains prevented regrowth after ALT removal and reincubation with sterile fresh media (Table 1, Figs. 1, 2, 3 and 4).

**Table 1:** Mean bacterial count recovered from the in vitro catheter model at different time-points

	1018			1022			1076			1098		
Atimicrobial regimen	t = 0	t = 72	t = 96	t = 0	t = 72	t = 96	t = 0	t = 72	t = 96	t = 0	t = 72	t = 96
Control	8,017	9,02	8,97	7,92	8,67	9,02	7,82	8,45	8,83	7,83	9,12	9,16
Teicoplanin	8,02	4,45*	6,72	7,09	4,03*	6,57	7,04	3,94*	7,44	7,89	3,84*	6,32
Teicoplanin + gentamycin	8,03	5,12*	7,01	7,43	4,5*	6,59	7,52	3,22*	6,67	7,78	2,64*	5,29
Teicoplanin + ethanol	7,94	1*	4,45*	7,85	1*	2,3*	7,27	1*	3,43	7,93	1*	4,02*
Teicoplanin + clarithromycin	7,95	1,89*	4,23*	7,86	1,23*	4,32*	7,29	1*	3,04	7,82	1,78*	5,38
Daptomycin	8,24	2,87*^,^ **	4,89*	7,33	1*^,^ **	3,77*	7,32	2,54*	5,48	7,82	3,62*	5,92
Daptomycin + gentamycin	8,12	2,03*^,^ **	5,67	7,51	3,69*	6,46	7,12	2,12*	4,92*	8,01	1,96*	5,01
Daptomycin + ethanol	7,98	1*	1*^,^ **	7,79	1*	1*^,^ **	7,027	1*	1*^,^ **	8,01	1*	1*^,^ **
Daptomycin + clarithromycin	8,34	1,45*	4,67*	7,95	1*	1*^,^ **	7,57	1*	1*^,^ **	7,83	1*	3,84*

Data expressed as Log_10_CFU/mL

T = 0: baseline. T = 72: end of antibiotic lock therapy. T = 96 h: after reincubation with sterile fresh media

*p < 0.05 versus control

**p < 0.05 vs teicoplanin (alone or in each combination compared against the same combination)

Despite full regrowth of all CoNS after ALT removal no emergence of resistance was detected against any of the antibiotics tested.

In our study we found ethanol and clarithromycin combinations more efficacious than monotherapy with either daptomycin or teicoplanin. Although monotherapy reached the limit of detection at 72 h against some strains, only daptomycin plus ethanol or clarithromycin prevented regrowth after ALT discontinuation.

Main objective of ALT is catheter salvage, allowing for reutilization of the catheter after discontinuation of ALT. If complete eradication has not been achieved, bacterial regrowth and resurgence of clinical infection are likely to occur. In our study, in an attempt of mimicking clinical practice, we introduced a 24 h dwell period with fresh media after ALT removal.

Although many authors have reported eradication of microorganisms after ALT with daptomycin, vancomycin or linezolid, because relapse after ALT was not evaluated, their results cannot be extrapolated to clinical practice [12, 15, 21]. In our study, daptomycin alone (against one strains) and some teicoplanin combinations achieved the limit of detection at 72 h but without complete eradication of biofilm. On the contrary, daptomycin plus clarithromycin (against two strains) and daptomycin plus ethanol (against all strains) maintained eradication after 24 h of ALT removal and re-incubation with fresh media, suggesting a potential role in salvage of infected catheters with a low rate of relapse.

A recent metanalysis support the use of ALT as adjunctive therapy in CLA-BSIs [11], however, a 20 % rate of relapse was observed in patients treated with ALT despite negative blood cultures drawn at the end of therapy. This high rate of relapse suggests the persistence of biofilm-embedded bacteria not detected immediately after end of therapy. Our results, negativization at 72 h, but regrowth after 24 h incubation with fresh media following ALT removal, corroborate what has been described, and might support the use of clarithromycin and ethanol combinations. Aumeran et al. reported the significant decrease of bacterial inoculum of MRSE in an in vitro model of subcutaneous injection port infection using daptomycin, vancomycin or ethanol, with a significant regrowth after discontinuation of ALT, similar to what we found [22]. Although they used a different methodology, the similarity of our results suggest that our hypothesis is true and relapse of CLA-BSIs is related to biofilm persistence despite negative blood-cultures drawn from the catheter after ALT.

Chaudhury et al. reported the efficacy of ethanol at 20, 40 and 80 %, in an in vitro study of MRSA and MRSE biofilm-associated infection [23]. Ethanol at any of the concentrations tested was more efficacious than vancomycin, ciprofloxacin and gentamicin in their model of biofilm-associated infection. Similarly Qu et al. reported that lock therapy with ethanol at 40 % was more efficacious than oxacillin, gentamicin, vancomycin, ciprofloxacin and rifampicin, alone or in combination [24]. We tested ethanol at 40, 60 and 80 % and found a significant decrease in Log_10_CFU/mL but without preventing regrowth after ALT discontinuation (data not shown). These preliminary results, similar to those reported by Raad et al. [25] that reported the inability of ethanol alone to eradicate an in vitro model of MRSA biofilm or Aumeran et al. [22], prompted us to explore antibiotic-ethanol combinations.

Similarly to what Raad et al. reported [25] antibiotic-ethanol combinations were significantly better than monotherapies and prevented regrowth after reincubation. They found that only ethanol (25 %) plus minocycline (3 mg/mL) and triple combination of ethanol, minocycline and EDTA (30 mg/mL) were able to eradicate biofilm and prevent regrowth in a modified Robbins device. In this silicone disk biofilm colonization model, although ethanol plus minocycline performed better than single-regimen therapies and eradicated biofilm, only triple therapy was able to prevent regrowth. In our study, the in vitro model of catheter infection was different, however we obtained very similar results, suggesting that ethanol plus antibiotics could eradicate biofilm and prevent regrowth after antibiotic lock removal.

Finally, Clarithromycin has shown to interact with bacterial biofilms through a non-bactericidal or bacteriostatic effect. It has been proven to inhibit the production of hexose, a basic component of staphylococcal biofilms [26]. Many authors have demonstrated this increased activity of clarithromycin combinations against staphylococcal biofilms despite no antibacterial activity of clarithromycin [27–30]. In this study, clarithromycin combinations outperformed single-regimen therapies. Daptomycin plus clarithromycin prevented regrowth of two strains, suggesting complete eradication of biofilm. On the other hand, Teicoplanin plus clarithromycin, although reduced the bacterial burden below the limit of detection, was not able to prevent regrowth, consistent with a reduced activity of teicoplanin against MRSE compared to that of daptomycin.

Our study has some limitations. Our protocol allowed for prolonged dwelling times, a situation that might limit its reproducibility in clinical practice. However, the fact that daptomycin plus ethanol and daptomycin plus clarithromycin (against two strain) were able to eradicate biofilm after only 72 h could avoid the need for this prolonged dwelling time. On the other hand, therapies longer than 3 days could improve our results. Second, we explored only four MRSE, so our results could not be extrapolated to all CLA-BSIs, however, as previously commented, other authors have reported similar results with ethanol or clarithromycin combinations with different strains [24, 25, 28, 30] which make plausible that our results could be generalized to MRSE and maybe, against MRSA, although the latter should be taken with caution based on paramount differences between CoNs and *Staphylococcus aureus*. Third, we did not evaluate the biofilm forming capability of the MRSE strains. Although is true that biofilm forming ability is different among different strains, we have selected four strains isolated from catheters of patients with documented CLA-BSIs. This clinical aspect together with the high Log_10_CFU/mL recovered prior to any antibiotic challenge suggests high biofilm forming ability.

Finally we tried to simulate daily clinical practice, so we chose commercially available drug powders and IV solutions for human use to make antibiotic lock solutions instead of on analytical powder. Although most other studies have used analytical drugs powder and so, our results are not completely comparable, we believe that our results would have been the same with analytical powder and, what is more important, they are more reproducible in clinical practice. Finally, we relied on the absence of precipitation as an equivalent of drug-stability; although there can be loss of potency without physical changes, it is widely accepted that absence of precipitation is an equivalent of stability and lock solutions can be use if no physical changes are documented [31–33].

## Conclusions

Our study provides strong evidence of the potential of daptomycin plus ethanol and daptomycin plus clarithromycin to eradicate biofilm and prevent regrowth after antibiotic lock removal. Although clinical trials should be performed, our results provide enough rationale supporting its use in antibiotic lock technique against MRSE.

## Methods

### Bacterial isolates

Four clinical isolates of methicillin resistant *Staphylococcus epidermidis* (MRSE); strains 1018, 1022, 1076, and 1098, recovered from catheters of patients with documented CLA-BSIs were selected from our collection.

### Antibiotics and media

Daptomycin (Cubicin® Novartis Inc. Spain) was generously provided by the manufacturer. Teicoplanin (Targocid® Marion-Merrel S.A. Spain), clarithromycin (Klacid® Abbot Laboratories S.A.), and gentamicin (Normon S.A. Spain) were commercially purchased as drug powder (Teicoplanin and clarithromycin) or as 20 mg/mL IV solution (gentamicin). Ethanol was commercially purchased as 70 % dilution (Lab. Reig Jofré. S.A. Spain). Drug powders were reconstituted following the CLSI guidelines [34].

Susceptibility testing was performed using e-test methodology but for DAP that was performed by microdilution following CLSI guidelines [34]. Mueller Hinton broth supplemented with 50 μg/ml calcium, and 12.5 μg/ml magnesium (SMHB) was used for susceptibility testing involving DAP.

Tryptic Soy Broth supplemented with 1 % glucose (TSBg) was used for biofilm growth.

To evaluate drug stability, each solution was incubated over 72 h, and then evaluated for physical compatibility by particulate formation, colour change, or gas evolution [31–33]. All drug-combinations maintained their initial conditions and were considered stable.

### In vitro model

Test organisms in TSBg at a starting inoculum of ~10^6^ CFU/mL were added to the lumen of an introcan Safety® 14 Ga × 2 in. catheter (Braun Medical Inc. USA). Each device was sealed and incubated for 24 h at 35 °C. After incubation, TSBg was removed and sterile drug-containing syringes were inserted into access port of the catheter. Each regimen (sufficient to fill the access port and full catheter) was slowly injected into each access port lumen and incubated at 37 °C. Antibiotics lock solutions were exchanged every 24 h for 72 h. After 72 h, last antibiotic lock solution was changed and catheters were filled again with fresh TSBg and incubated for another 24 h to reproduce reutilization after ALT.

Catheters were removed at 0, 4, 8, 24, 48, 72 and 96 h. The lock solution was removed and whole catheters were placed in a sterile tube filled with 10 mL of saline, vortexed, sonicated and flushed thoroughly. The bacterial suspension was then diluted and drop-plated on Tryptic soy agar (TSA) for bacterial enumeration. The limit of detection of this method of colony count determination was 2 Log extended to 1 Log by vacuum filtration.

Bactericidal activity was defined as > 3 Log kill reduction from initial inoculum at any time point, and emergence of resistance was assessed by E-test method.

Experiments were performed in triplicate to account for biological variability. Results are expressed as mean log10 reduction.

### Lock solutions evaluated

Teicoplanin at 5 mg/mL and daptomycin at 5 mg/mL (diluted in ringer lactate), both alone and in combination with gentamicin at 2.5 mg/mL, clarithromycin at 5 mg/mL and ethanol at 35 % were compared. For combination regimens, final concentrations were tailored to be the same as for monotherapies. As daptomycin activity is calcium-dependent, gentamicin and clarithromycin were diluted in ringer lactate when combined with daptomycin and calcium was added to ringer lactate when daptomycin was combined with ethanol, to maintain the same calcium concentration in every daptomycin lock solution. Phosphate buffered saline (PBS) was used as control.

### Statistical analysis

Time to achieve 99.9 % kill (T_99.9_) was determined by linear regression (if r^2^ ≤ 0.95) or visual inspection.

Changes in bacterial CFU/mL were compared by ANOVA with Tukey’s post-hoc test. A *p* value < 0.05 was considered significant.

## Abbreviations

ALT: Antibiotic lock technique
DAP: Daptomycin
TEC: Teicoplanin
GM: Gentamicin
CLA: Clarithromycin
CLA-BSIs: Central line bloodstream infections
CoNS: Coagulase negative *staphylococci*
MRSE: Methicillin resistant *Staphylococcus epidermidis*
CLSI: Clinical laboratory standard institute
TSBg: Tryptic soy broth supplemented with 1 % glucose
TSA: Tryptic soy agar
PBS: Phosphate buffered saline
CFU: Colony forming units
MIC: Minimum inhibitory concentration
MRSA: Methicillin-resistant *Staphylococcus aureus*

## References

[1] Rosenthal VD, Maki DG, Mehta Y, Leblebicioglu H, Memish ZA, Al-Mousa HH (2014). International Nosocomial Infection Control Consortium (INICC) report, data summary of 43 countries for 2007–2012. Device-associated module. Am J Infect Control.

[2] Hidron AI, Edwards JR, Patel J, Horan TC, Sievert DM, Pollock DA (2008). NHSN annual update: antimicrobial-resistant pathogens associated with healthcare-associated infections: annual summary of data reported to the National Healthcare Safety Network at the Centers for Disease Control and Prevention, 2006–2007. Infect Control Hosp Epidemiol.

[3] Strasheim W, Kock MM, Ueckermann V, Hoosien E, Dreyer AW, Ehlers MM (2015). Surveillance of catheter-related infections: the supplementary role of the microbiology laboratory. BMC Infect Dis.

[4] Weber DJ, Rutala WA (2011). Central line-associated bloodstream infections: prevention and management. Infect Dis Clin North Am.

[5] Doshi RK, Patel G, Mackay R, Wallach F (2009). Healthcare-associated infections: epidemiology, prevention, and therapy. Mt Sinai J Med.

[6] Simon A, Bode U, Beutel K (2006). Diagnosis and treatment of catheter-related infections in paediatric oncology: an update. Clin Microbiol Infect.

[7] Mermel LA, Allon M, Bouza E, Craven DE, Flynn P, O’Grady NP (2009). Clinical practice guidelines for the diagnosis and management of intravascular catheter-related infection: 2009 Update by the Infectious Diseases Society of America. Clin Infect Dis.

[8] O’Grady NP, Chertow DS (2011). Managing bloodstream infections in patients who have short-term central venous catheters. Cleve Clin J Med.

[9] Fernandez-Hidalgo N, Almirante B (2014). Antibiotic-lock therapy: a clinical viewpoint. Expert Rev Anti Infect Ther.

[10] Lebeaux D, Fernandez-Hidalgo N, Chauhan A, Lee S, Ghigo JM, Almirante B (2014). Management of infections related to totally implantable venous-access ports: challenges and perspectives. Lancet Infect Dis.

[11] O’Horo JC, Silva GL, Safdar N (2011). Anti-infective locks for treatment of central line-associated bloodstream infection: a systematic review and meta-analysis. Am J Nephrol.

[12] LaPlante KL, Mermel LA (2007). In vitro activity of daptomycin and vancomycin lock solutions on staphylococcal biofilms in a central venous catheter model. Nephrol Dial Transplant.

[13] Balestrino D, Souweine B, Charbonnel N, Lautrette A, Aumeran C, Traore O (2009). Eradication of microorganisms embedded in biofilm by an ethanol-based catheter lock solution. Nephrol Dial Transplant.

[14] Bookstaver PB, Williamson JC, Tucker BK, Raad II, Sherertz RJ (2009). Activity of novel antibiotic lock solutions in a model against isolates of catheter-related bloodstream infections. Ann Pharmacother.

[15] Sherertz RJ, Boger MS, Collins CA, Mason L, Raad II (2006). Comparative in vitro efficacies of various catheter lock solutions. Antimicrob Agents Chemother.

[16] Van Praagh AD, Li T, Zhang S, Arya A, Chen L, Zhang XX (2011). Daptomycin antibiotic lock therapy in a rat model of staphylococcal central venous catheter biofilm infections. Antimicrob Agents Chemother.

[17] Raad I, Hanna H, Jiang Y, Dvorak T, Reitzel R, Chaiban G (2007). Comparative activities of daptomycin, linezolid, and tigecycline against catheter-related methicillin-resistant Staphylococcus bacteremic isolates embedded in biofilm. Antimicrob Agents Chemother.

[18] Oncu S, Sakarya S (2005). Comparison of different doses of vancomycin andteicoplanin lock solutions in catheters colonized with Staphylococcus epidermidis: An in vitro, blinded, antibiotic lock study. Curr Ther Res Clin Exp.

[19] Del Pozo JL, Garcia Cenoz M, Hernaez S, Martinez A, Serrera A, Aguinaga A (2009). Effectiveness of teicoplanin versus vancomycin lock therapy in the treatment of port-related coagulase-negative staphylococci bacteraemia: a prospective case-series analysis. Int J Antimicrob Agents.

[20] Meije Y, Almirante B, Del Pozo JL, Martin MT, Fernandez-Hidalgo N, Shan A (2014). Daptomycin is effective as antibiotic-lock therapy in a model of Staphylococcus aureus catheter-related infection. J Infect.

[21] Giacometti A, Cirioni O, Ghiselli R, Orlando F, Mocchegiani F, Silvestri C (2005). Comparative efficacies of quinupristin-dalfopristin, linezolid, vancomycin, and ciprofloxacin in treatment, using the antibiotic-lock technique, of experimental catheter-related infection due to Staphylococcus aureus. Antimicrob Agents Chemother.

[22] Aumeran C, Guyot P, Boisnoir M, Robin-Hennequin C, Vidal M, Forestier C (2013). Activity of ethanol and daptomycin lock on biofilm generated by an in vitro dynamic model using real subcutaneous injection ports. Eur J Clin Microbiol Infect Dis.

[23] Chaudhury A, Rangineni J, Venkatramana B. Catheter lock technique: in vitro efficacy of ethanol for eradication of methicillin-resistant staphylococcal biofilm compared with other agents. FEMS Immunol Med Microbiol. 2012. 10.1111/j.1574-695X.2012.00950.x 22380476

[24] Qu Y, Istivan TS, Daley AJ, Rouch DA, Deighton MA (2009). Comparison of various antimicrobial agents as catheter lock solutions: preference for ethanol in eradication of coagulase-negative staphylococcal biofilms. J Med Microbiol.

[25] Raad I, Hanna H, Dvorak T, Chaiban G, Hachem R (2007). Optimal antimicrobial catheter lock solution, using different combinations of minocycline, EDTA, and 25-percent ethanol, rapidly eradicates organisms embedded in biofilm. Antimicrob Agents Chemother.

[26] Yasuda H, Ajiki Y, Koga T, Yokota T (1994). Interaction between clarithromycin and biofilms formed by Staphylococcus-epidermidis. Antimicrob Agents Chemother.

[27] Furuhata M, Iwamura M, Baba S, Inoue M (2003). Combined effect of clarithromycin and imipenem/cilastatin against urinary biofilm infection after pyeloplasty. Int J Urol.

[28] Fujimura S, Sato T, Kikuchi T, Zaini J, Gomi K, Watanabe A (2009). Efficacy of clarithromycin plus vancomycin in mice with implant-related infection caused by biofilm-forming Staphylococcus aureus. J Orthop Sci.

[29] Fujimura S, Sato T, Mikami T, Kikuchi T, Gomi K, Watanabe A (2008). Combined efficacy of clarithromycin plus cefazolin or vancomycin against Staphylococcus aureus biofilms formed on titanium medical devices. Int J Antimicrob Agents.

[30] Parra-Ruiz J, Vidaillac C, Rose WE, Rybak MJ (2010). Activities of high-dose daptomycin, vancomycin, and moxifloxacin alone or in combination with clarithromycin or rifampin in a novel in vitro model of Staphylococcus aureus biofilm. Antimicrob Agents Chemother.

[31] Anthony TU, Rubin LG (1999). Stability of antibiotics used for antibiotic-lock treatment of infections of implantable venous devices (ports). Antimicrob Agents Chemother.

[32] Fernandez-Hidalgo N, Almirante B, Calleja R, Ruiz I, Planes AM, Rodriguez D (2006). Antibiotic-lock therapy for long-term intravascular catheter-related bacteraemia: results of an open, non-comparative study. J Antimicrob Chemother.

[33] Droste JC, Jeraj HA, MacDonald A, Farrington K (2003). Stability and in vitro efficacy of antibiotic-heparin lock solutions potentially useful for treatment of central venous catheter-related sepsis. J Antimicrob Chemother.

[34] Clinical and Laboratory Standards Institute (2008). Methods for dilution antimicrobial susceptibility tests for bacteria that grow aerobically; approved standard.

